# Metastatic Lung Adenocarcinoma With Occult Involvement of Gluteal Muscles as the Sole Site of Distant Metastases

**DOI:** 10.7759/cureus.9826

**Published:** 2020-08-18

**Authors:** Syed Ather Hussain, Dharmini Manogna, Joel Shapiro

**Affiliations:** 1 Internal Medicine, Rochester General Hospital, Rochester, USA; 2 Pathology, Rochester General Hospital, Rochester, USA

**Keywords:** metastatic lung adenocarcinoma, occult metastases, gluteal muscles

## Abstract

Lung cancer metastases to soft tissues are rarely reported in the literature. In this report, we discuss a case of a 59-year-old female who presented with worsening shortness of breath for over five months. A CT scan of the chest revealed right upper lobe mass and ipsilateral mediastinal adenopathy. An endo-bronchial ultrasound (EBUS)-guided biopsy of the involved lymph nodes revealed cellular features consistent with lung adenocarcinoma. MRI of the brain was negative for metastases; however, a positron emission testing (PET) scan showed fluorodeoxyglucose (FDG)-avid nodules in the soft tissues of the bilateral buttocks. Tissue biopsy of the buttock lesions confirmed metastases of lung origin. To the best of our knowledge, this is the first case report of metastatic lung adenocarcinoma with occult involvement of the gluteal muscles as the sole site of distant metastasis.

## Introduction

Lung cancer is the leading cause of cancer-related deaths [1]. Approximately 50% of lung cancer cases are found to be metastatic at the time of the diagnosis [1]. Lung cancer can metastasize to almost any organ including the liver (33-40%), adrenal glands (18-38%), brain (15-43%), bone (19-33%), kidney (16-23%), and abdominal lymph nodes (29%) [1]. Metastases to soft tissues, which include skeletal muscle, subcutaneous tissues, and skin have been scarcely reported. In the literature, two cases of squamous cell lung carcinoma, one with occult involvement and the other with gross involvement of the gluteal muscles, have been reported [2,3]. A case of metastatic lung adenocarcinoma with a palpable thigh nodule has also been reported [4]. In this report, we present a case of metastatic lung adenocarcinoma with occult involvement of the gluteal muscles as the sole site of distant metastasis.

## Case presentation

A 59-year-old female with a history of smoking presented with complaints of gradually worsening shortness of breath over five months. She denied chest pain, cough, and hemoptysis. She had a decreased appetite but no weight loss. She also reported dull right shoulder pain for three months. She had smoked one pack of cigarettes per day for 40 years but had quit 17 months ago. She had worked in the printing business for 23 years. She had no known history of exposure to tuberculosis or asbestosis. Family history was significant for squamous cell lung cancer in her sister at the age of 32 years.

On examination, blood pressure was 137/62 mmHg, heart rate was 81 beats/min, and the temperature was 36.7 °C. She did not have any palpable cervical, supraclavicular, or axillary adenopathy. Heart sounds were regular. Lung sounds were clear bilaterally. The abdomen was non-distended with no hepatosplenomegaly. No rashes, pedal edema, or soft tissue nodules were appreciated. Initial lab work revealed a white blood cell count of 8.3 x 10^3^/μL, hemoglobin of 9.5 g/dL, and platelet count of 509 x 10^3^/μL. Basic metabolic panel revealed serum sodium of 137 mEq/L, serum potassium of 4.2 mEq/L, blood urea nitrogen of 8 mg/dL, and creatinine of 0.5 mg/dL.

**Figure 1:**
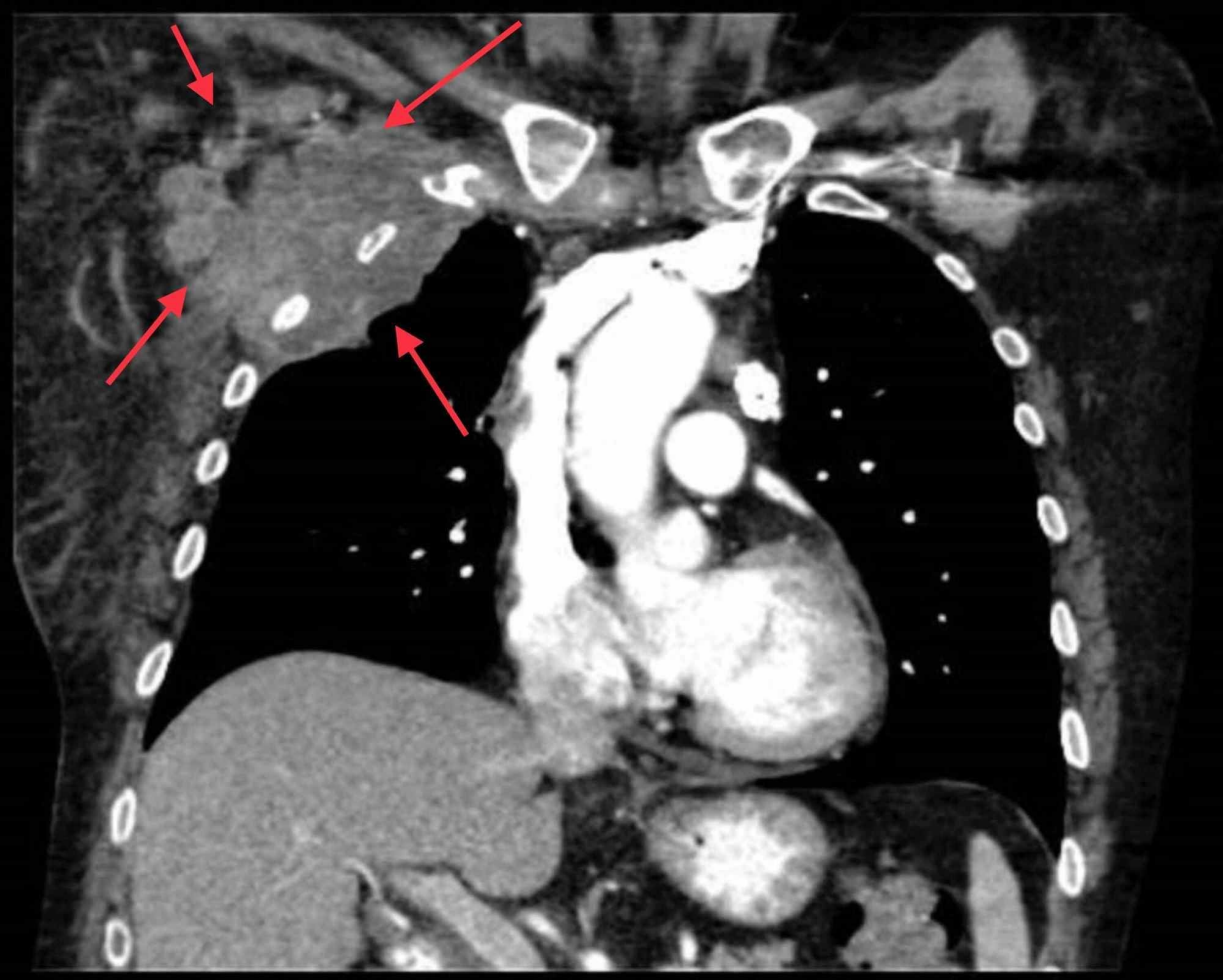
Coronal CT scan The arrows show large mass along the right chest wall measuring 8.6 x 8 cm with adjacent conglomerate adenopathy. There is associated destruction of right ribs 2 and 3 with pathologic fractures CT: computed tomography

**Figure 2:**
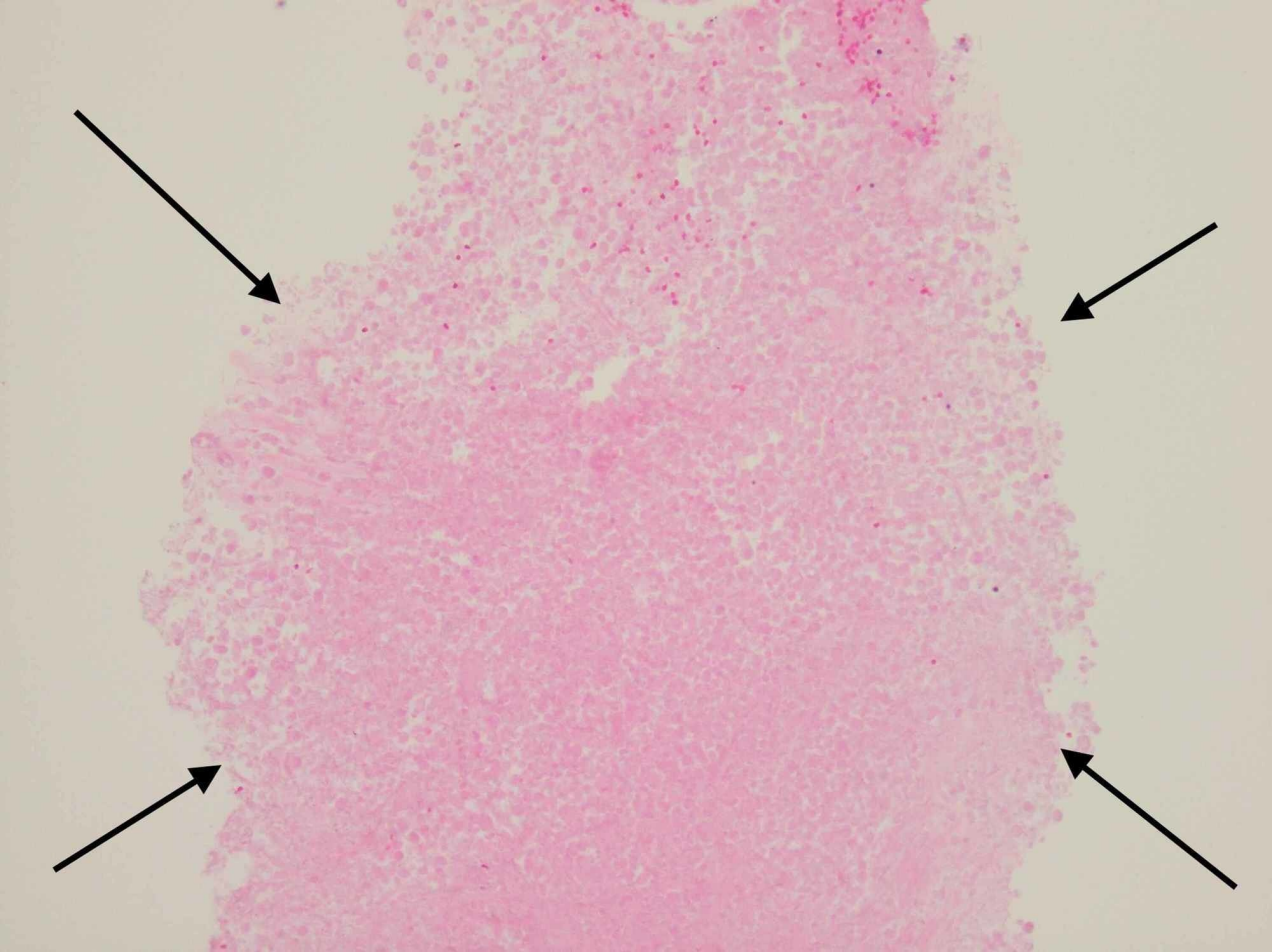
Lung biopsy The arrows show necrosis and ghost cells

**Figure 3:**
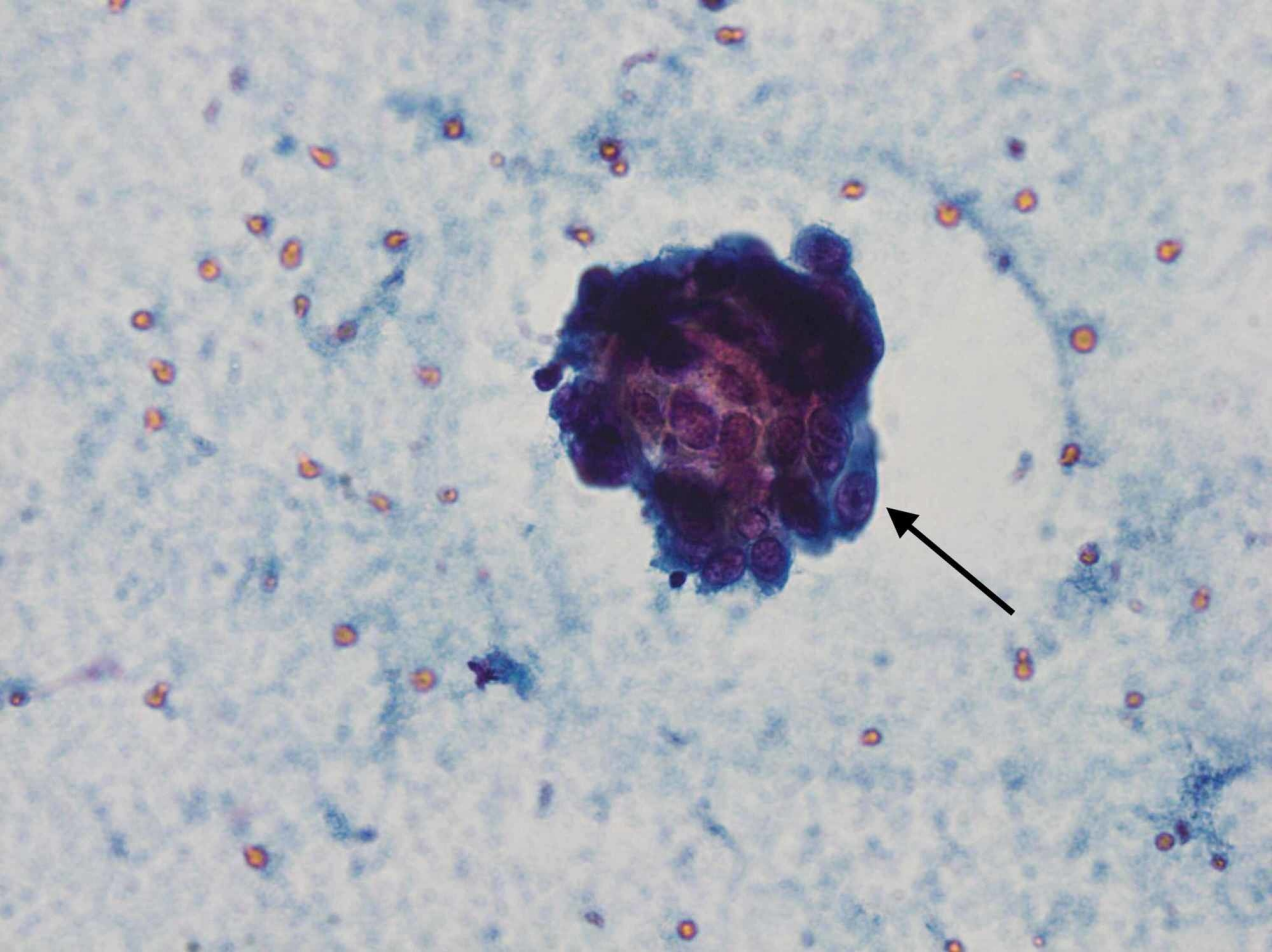
Right subcarinal lymph node biopsy The arrow shows enlarged, hyperchromatic tumor cells with coarse chromatin and prominent nucleoli. Cells were visualized with Pap stain

**Figure 4:**
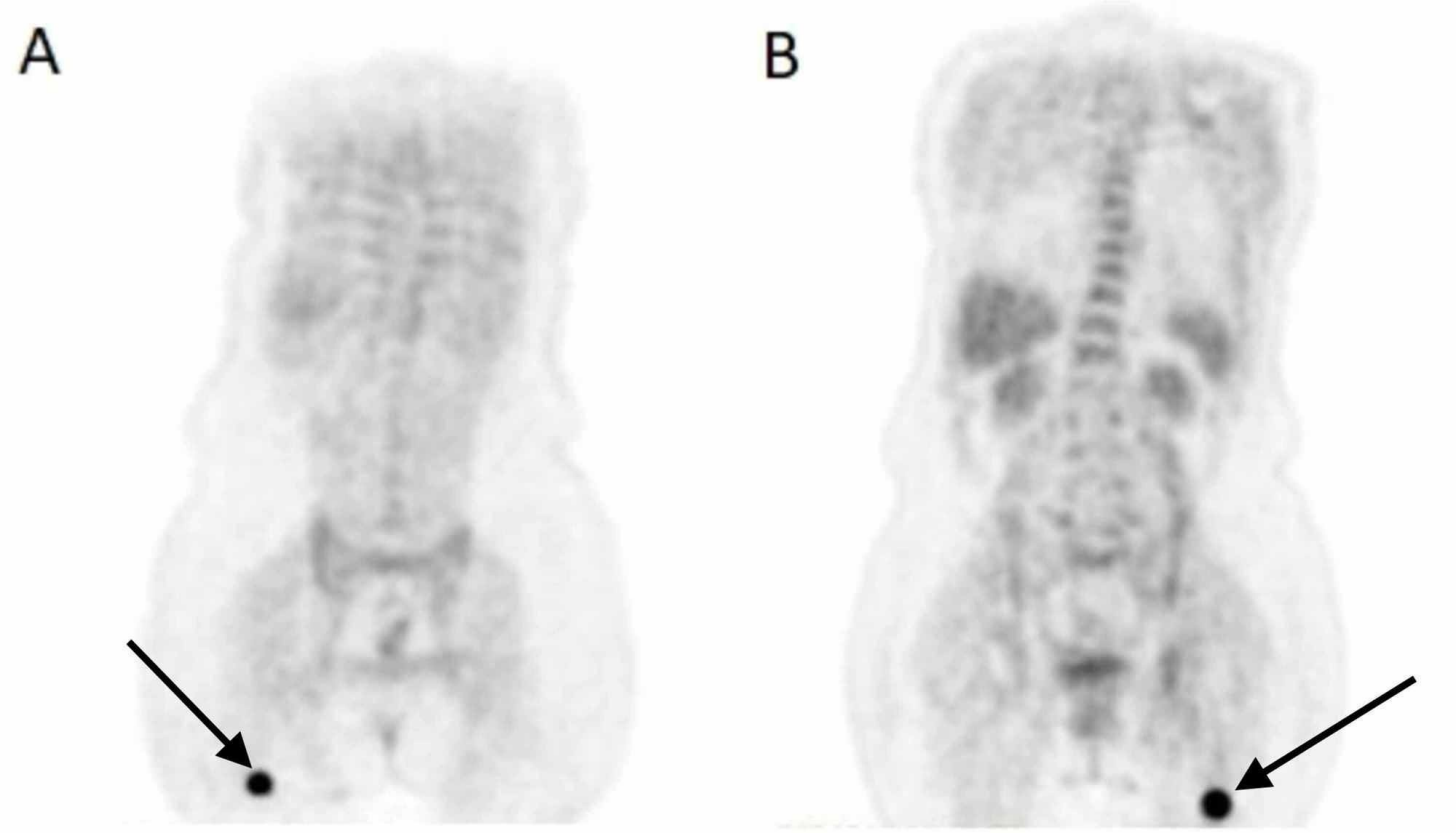
Positron emission tomography scan The arrows show right (A) and left (B) metastatic gluteal occult nodules

**Figure 5:**
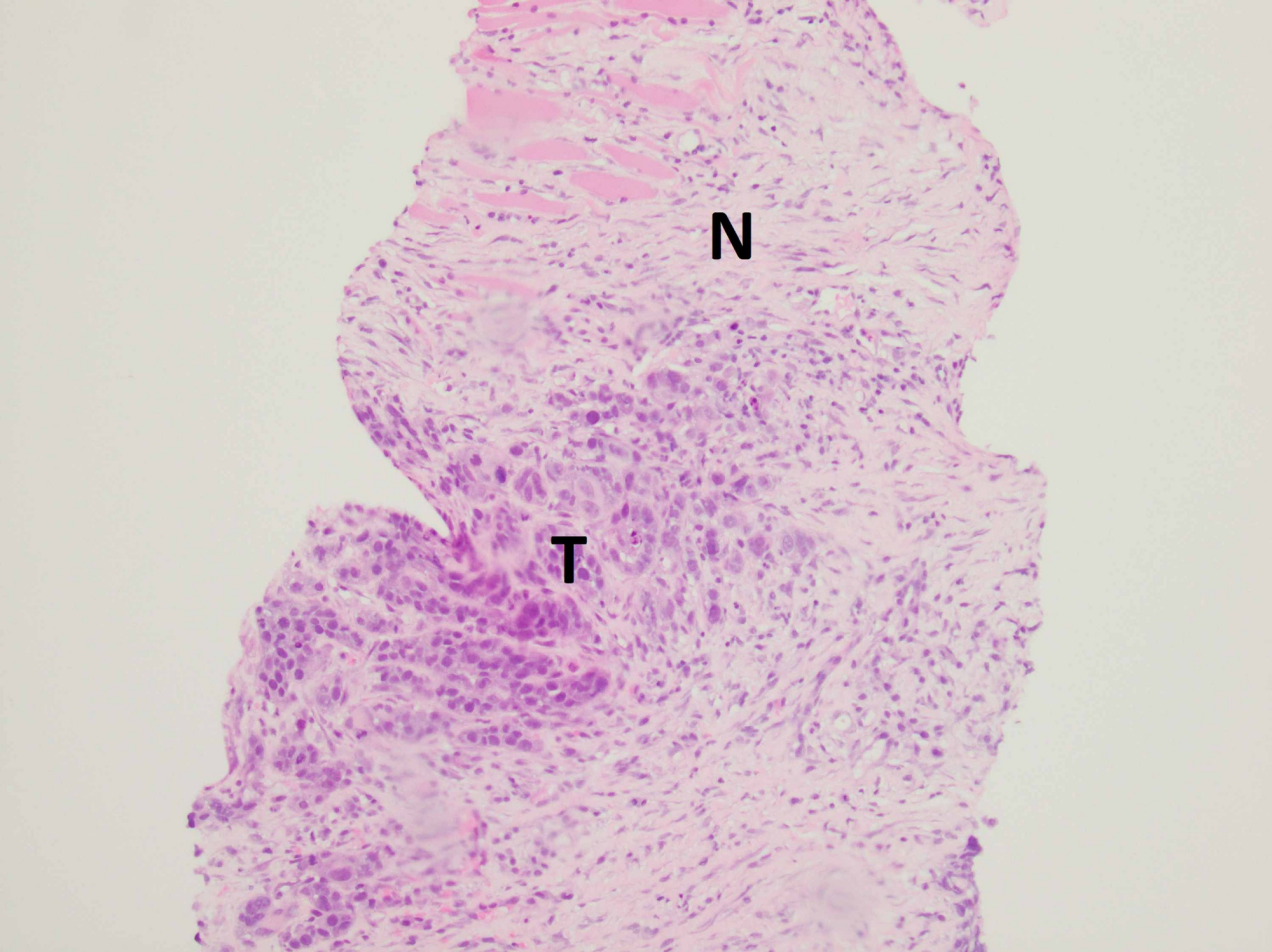
Gluteal biopsy The image shows normal striated skeletal muscle adjacent to glandular structures representative of metastatic lung adenocarcinoma. Region of the tumor is marked by letter "T" and normal muscle tissue is marked by letter "N"

A CT scan of the chest was done, which showed right upper lobe mass and ipsilateral mediastinal adenopathy (Figure 1). Biopsy of the lung mass showed necrotic tissue, which was non-diagnostic (Figure 2). Subsequently, an endo-bronchial ultrasound-guided fine-needle aspiration (EBUS-FNA) biopsy was performed, which revealed enlarged, hyperchromatic cells with coarse chromatin and prominent nucleoli in the right subcarinal and paratracheal lymph nodes (Figure 3). Tumor markers were positive for cytokeratin-7 (CK7), thyroid transcription factor 1 (TTF-1), and napsin A, consistent with lung adenocarcinoma. Mutation analysis was negative for BRAF V600E, epidermal growth factor receptor (EGFR), anaplastic lymphoma kinase (ALK) 2p23, and ROS1 6q22 gene arrangement. The programmed death ligand-1 (PD-L1) tumor proportion score was 100%. At this point, given that she had at least stage IIIA disease and a fairly bulky tumor, she was deemed to not be a candidate for surgery and neoadjuvant chemotherapy. MRI of the brain was negative for metastases; however, a positron emission tomography (PET) scan showed significant fluorodeoxyglucose (FDG) avidity in the right upper lobe lung mass with direct extension through the chest wall along with the involvement of the ipsilateral mediastinal, neck, and axillary lymph nodes. It was thought that this could be due to lymphatic spread secondary to chest wall invasion, which would classify this malignancy as stage III disease. Notably, the PET scan also showed FDG-avid nodules in the soft tissues of the bilateral buttocks (Figure 4). Thereafter, these lesions were biopsied and revealed metastatic lung adenocarcinoma (Figure 5). Palliative treatment with pembrolizumab, cisplatin, and pemetrexed was initiated. Unfortunately, her disease continued to progress and ultimately she died from complications five months after diagnosis.

## Discussion

Our patient not only had a positive family history but also occupational exposure to ink mist, which increased her risk of developing lung cancer. A nested case-control study conducted in the United Kingdom has shown that the duration of work in a letterpress machine room was positively associated with the risk of lung cancer. Benzoapyrene, a known human carcinogen, is believed to be the causative agent in such cases as it is present in high concentrations in the atmosphere of a printing room [5].

Metastases to muscle are uncommon with a reported prevalence of only 0-0.8% of muscular metastasis among lung cancer patients [4]. The effect of lactic acid production, changeable blood flow, variable tissue oxygen tensions, beta-adrenergic stimulation, and tissue defense mechanisms might prevent seeding of the tumor metastases [6]. Trauma to the muscle can disrupt the local physiology via the release of growth factors or fibrin clots and hence allow entrapment of circulating tumor cells [7]. Spread to the muscle usually occurs hematogenously; however, other possible mechanisms include spread through venous plexuses, lymphatics, endo-bronchial and direct spread [2].

One study discovered that patients with lung adenocarcinoma or large-cell carcinoma were at a higher risk for extra-thoracic metastases when compared to patients with squamous cell lung carcinoma [8]. The most commonly reported locations are the back, chest wall, and abdomen [8]. Lung cancer soft tissue metastases usually present as rapidly growing solitary or multiple painless nodules with a diameter between 5 mm to 10 cm covered with normal skin [6]. Occasionally, exudative or ulcerative lesions may also be seen [8]. Interestingly, our patient had no subjective discomfort or external lesions raising suspicion of soft tissue metastases. The differential diagnosis for soft tissue metastases is broad and includes primary soft tissue sarcomas, primary muscle lymphomas, and benign diseases such as muscle hemangiomas, intramuscular gangliomas, and myxomas [1]. Ischiogluteal bursitis is also commonly seen in cancer patients and can mimic muscle metastases [1]. A thorough physical examination is of paramount importance as soft tissue metastases can have prognostic implications. Physical exam in our patient was unrevealing, and the initial plan was to offer concurrent chemoradiation therapy followed by adjuvant immunotherapy. However, with positive gluteal nodules seen on PET scan and biopsy showing metastatic adenocarcinoma most consistent with lung origin, her disease qualified as stage IV and only palliative therapy could be offered.

The choice of treatment is based on multiple factors including comorbidities, histopathology, and mutations [2]. Treatment options include palliative external radiation therapy and combination chemotherapy; and combination chemotherapy, targeted therapy, and immunotherapy [2]. Prompt diagnosis and treatment may prolong survival.

## Conclusions

We presented a rare case of lung adenocarcinoma with occult metastases to the gluteal muscles as the sole site of distant spread. PET scan and tissue biopsy confirmed metastatic disease to the gluteal muscles, which was treated with pembrolizumab, cisplatin, and pemetrexed. PET scan can be instrumental in the identification of occult lesions. A low threshold should be kept for performing a biopsy to distinguish soft tissue metastases from other pathologies. Timely initiation of chemotherapy, immunotherapy, and targeted therapy may help in prolonging survival rates.
